# Seesaw Effect Between COVID-19 and Influenza From 2020 to 2023 in World Health Organization Regions: Correlation Analysis

**DOI:** 10.2196/44970

**Published:** 2023-06-12

**Authors:** Qing Wang, Mengmeng Jia, Mingyue Jiang, Wei Liu, Jin Yang, Peixi Dai, Yanxia Sun, Jie Qian, Weizhong Yang, Luzhao Feng

**Affiliations:** 1 School of Population Medicine and Public Health Chinese Academy of Medical Sciences & Peking Union Medical College Beijing China; 2 Department of Statistics Yunnan University Kunming China; 3 Division of Infectious Diseases Chinese Center for Disease Control and Prevention Beijing China

**Keywords:** COVID-19, influenza, negative correlation, seesaw effect, respiratory infectious disease, epidemiological trends

## Abstract

**Background:**

Seasonal influenza activity showed a sharp decline in activity at the beginning of the emergence of COVID-19. Whether there is an epidemiological correlation between the dynamic of these 2 respiratory infectious diseases and their future trends needs to be explored.

**Objective:**

We aimed to assess the correlation between COVID-19 and influenza activity and estimate later epidemiological trends.

**Methods:**

We retrospectively described the dynamics of COVID-19 and influenza in 6 World Health Organization (WHO) regions from January 2020 to March 2023 and used the long short-term memory machine learning model to learn potential patterns in previously observed activity and predict trends for the following 16 weeks. Finally, we used Spearman correlation coefficients to assess the past and future epidemiological correlation between these 2 respiratory infectious diseases.

**Results:**

With the emergence of the original strain of SARS-CoV-2 and other variants, influenza activity stayed below 10% for more than 1 year in the 6 WHO regions. Subsequently, it gradually rose as Delta activity dropped, but still peaked below Delta. During the Omicron pandemic and the following period, the activity of each disease increased as the other decreased, alternating in dominance more than once, with each alternation lasting for 3 to 4 months. Correlation analysis showed that COVID-19 and influenza activity presented a predominantly negative correlation, with coefficients above –0.3 in WHO regions, especially during the Omicron pandemic and the following estimated period. The diseases had a transient positive correlation in the European region of the WHO and the Western Pacific region of the WHO when multiple dominant strains created a mixed pandemic.

**Conclusions:**

Influenza activity and past seasonal epidemiological patterns were shaken by the COVID-19 pandemic. The activity of these diseases was moderately or greater than moderately inversely correlated, and they suppressed and competed with each other, showing a seesaw effect. In the postpandemic era, this seesaw trend may be more prominent, suggesting the possibility of using one disease as an early warning signal for the other when making future estimates and conducting optimized annual vaccine campaigns.

## Introduction

Influenza was primarily characterized as a seasonal epidemic before the COVID-19 pandemic. Stringent public health and social interventions (PHSMs) were implemented in early 2020 to contain the spread of SARS-CoV-2, and influenza activity also declined [1-3]. However, during the latest winter and spring, influenza re-emerged and had a three-year-high level of activity in China [4] and other countries [5] at the same time that there was reduced virulence of Omicron and easing of PHSMs. Under the same natural conditions and PHSMs in a region, there seems to be a subtle relationship between these 2 respiratory infections. For example, they have been found to cocirculate and to coinfect people [6], but most study subjects have been COVID-19 patients [6,7]. General population-based epidemiological patterns and regularities have not yet been fully explored.

Member countries of the World Health Organization (WHO) regions update influenza activity data weekly in the FluNet, a web-based global influenza virology surveillance tool first introduced in 1997 [8]. The WHO divides member countries into 6 regions based on their location, including the African region (AFRO), Eastern Mediterranean region (EMRO), European region (EURO), Americas region (AMRO), Southeast Asian region (SEARO), and Western Pacific region (WPRO) [9]. Since January 2020, a subset of member countries in each region have also reported their national test positivity rates for SARS-CoV-2 on a weekly basis. Influenza sentinel surveillance systems were leveraged to integrate SARS-CoV-2 testing in specimens from influenza surveillance sources. The genomic sequencing of SARS-CoV-2 from representative and systematically sourced sentinel specimens has been expedited to monitor the trends and prevalence (ie, proportions) of existing and emerging circulating and cocirculating genetic variants (ie, clades) and to improve the geographic and demographic representativeness and timeliness of SARS-CoV-2 genetic-sequence data in publicly accessible databases to inform PHSMs [10].

Most studies have conducted epidemiological analyses and estimation of COVID-19 or influenza by leveraging infectious disease dynamics models [11-13] (eg, the susceptible-exposed-infected-removed model), but few have predicted trends based on longer time series and machine learning during the past 3 years. These models help tackle multimodal data and are increasingly being used in auxiliary diagnosis and other medical areas [14,15]. They are composed of different networks with various connections, referred to as neural networks or neural-like networks, to achieve automated learning and prediction by mimicking the neural network construction of the human brain. This method is highly adaptable and can adjust itself to diverse data sets, thus adapting itself to different application scenarios. Secondly, deep learning avoids human specification by automatically extracting data features and processing them. This is highly scalable and can improve simulation performance by increasing the network layers and nodes to cope with larger-scale data and more complex problems [16]. Therefore, more learning models could be introduced into infectious disease surveillance and studies.

In this study, we examined global changes in COVID-19 and influenza activity since 2020, aiming to assess the correlation between COVID-19 and influenza activity and estimate upcoming trends with the help of a deep learning model. The findings may provide a theoretical reference for the epidemiological patterns of these 2 respiratory infectious diseases and insights for future interventions.

## Methods

### Summary of the Study Design

In this study, we retrospectively described the dynamics of COVID-19 and influenza in the 6 WHO regions from January 2020 to March 2023 and used a neural network model to learn potential patterns of previously observed activity to predict trends for the next 16 weeks. Finally, based on an epidemiological perspective, Spearman correlation coefficients were used to assess the past and future correlations. The analysis was performed using Python (version 3.9.6; Python Software Foundation).

### Data Source

The test positivity rate was used as an accurate indicator of disease activity; this method used the number of positive tests as the numerator and the specimens processed as the denominator. We extracted the weekly test positivity rates for influenza and SARS-CoV-2 from reports to FluNet between January 12, 2020, and March 26, 2023 [10], covering the AFRO, EMRO, EURO, AMRO, SEARO, and WPRO. The detailed raw data names and sources are presented in Multimedia Appendix 1, Table S1.

### Model Construction

**Figure 1:**
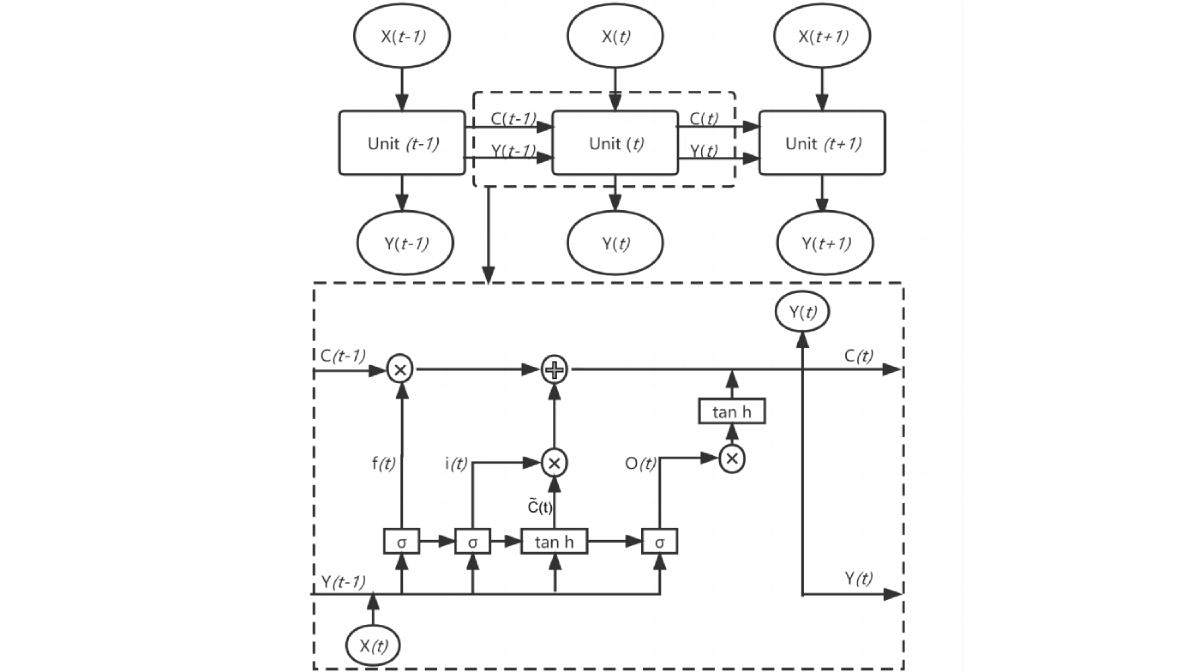
Schematic diagram of the long short-term memory neural network framework. The neural network consists of many units. The dashed box shows one of the unit structures, containing the forgetting gate, the input gate, and the output gate control system. The circles intersected by arrows in the dotted box denote multiplication or addition corresponding to the formula for the method.

Based on the 3-year activity of COVID-19 and influenza, we used a long short-term memory (LSTM) machine learning model to estimate trends in the following 16 weeks. LSTM is widely applied to stock and sales forecasting, specializing in a unique memory cell structure that can process long time-series information more effectively than other net models. It can be trained directly on the series without smoothness requirements, reducing the need for artificial intervention and enhancing objectivity [16]. The LSTM transforms the traditional neurons of a neural network into store cells, which are capable of storing and transmitting the hidden information in a time series (Figure 1).

The LSTM converts conventional neurons into a storage unit containing a matrix of 3 gating devices: input gate, forgetting gate, and output gate. These combine to control the data information flow and store the hidden information in the time series [17], hence solving the gradient disappearance [18,19] (Figure 1).

First, the forgetting gate filters the information in the unit C_t–1_ at the previous moment t–1, and how much of its C_t–1_ information is retained depends on the forget gate f_t_ that has the range [0, 1]; f_t_ is set as 1 or 0 and in between, with the information of C_t–1_ corresponding to all retained, all forgotten, and partially forgotten proportions, respectively. In the forgetting gate unit equation (equation 1), let W_f_, X_t_, y_t–1_, and b_f_ denote the weight of the forgetting gate, the input at time t, the hidden state at time t–1, and the bias of the forget gate. The sigmoid activation function is σ (equation 2).

Second, the input gate represents the information acquired at the moment t. It determines how much information from the input X_t_ at the moment t can be saved in the unit C_t_ (equations 3 and 4). W_i_ and W_c_ are the weights of the input gates. The biases of the input gates are b_i_ and b_c_, and tanh is the activation function (equation 5).

Third, the update of the unit status C_t_ (equation 6) depends on two parts: One is the product of the unit status C_t_ at the moment t–1 and the forget gate f_t_, namely the retained information, and the other part is the product of the input unit status C ~_t_ at the present moment t and the input gate i_t_, namely the new information obtained. C ~_t_ is the input node at time t.

Fourth, the output gate is used to output the hidden status y_t_ at the moment t (equations 7 and 8). W_o_ and b_o_ are the respective weights and biases of the output gates.

### Model Performance Evaluation and Selection of Optimal Parameters

To enhance the model prediction accuracy and scalability, we used the mean absolute percentage error and root mean squared error as evaluation indicators to determine the optimal proportion of training set and test set, as well as step size, layers, nodes, and algorithms [20,21], for different regions. The selection process is detailed in Multimedia Appendix 1, Tables S2-S6.




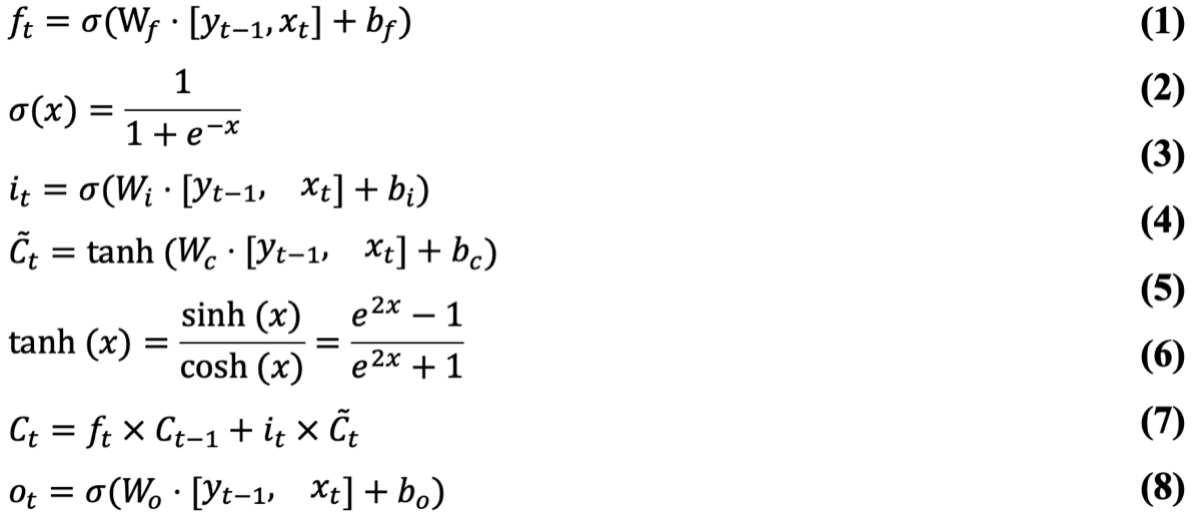




### Correlation Analysis

We referred to the WHO list of the currently circulating variants of concern of SARS-CoV-2 [22] and the study objectives to uniformly divide all regions into 5 time periods to analyze the correlations: these periods covered the original strain (January 2020 to November 2020), cocirculation of a multivariant strain (referred to as “others”; December 2020 to April 2021), Delta (May 2021 to November 2021), Omicron (November 2021 to March 2023), and the period estimated by the models (March 2023 to July 2023). The starting and ending dates of variant pandemics in different regions differed slightly. In the 6 WHO regions, the Spearman nonparametric analysis of COVID-19 and influenza activity was conducted sequentially to identify whether the epidemiological patterns were correlated across the 5 time periods mentioned above. The correlation analysis was not intended to reveal causality but rather to identify and explain significant trends.

### Ethical Statement

As only published data were used for this study, ethical approval was not necessary.

## Results

### Description of Past Trends

Before the Delta variant emerged, the activity of the original strain and other variants of SARS-CoV-2 was higher than that of influenza in the 6 regions. Among the regions, SARS-CoV-2 activity was observed in AFRO, EMRO, and AMRO at a high level (peaking at over 30%), and influenza activity was at a low level (peaking at about less than 10%). Both fluctuated with low activity levels (less than 15% overall), but influenza activity was relatively lower in SEARO, WPRO, and EURO.

Following the emergence of Delta as the globally dominant strain, influenza activity began to increase in WHO regions, but in the AMRO and EURO, activity was less than 10% and remained below that of Delta. In the other 4 WHO regions, Delta activity reached a peak (over 30%) and then declined to below 10%, and the decline was accompanied by a step-up in influenza activity to 10% to 20% until the advent of Omicron.

**Figure 2:**
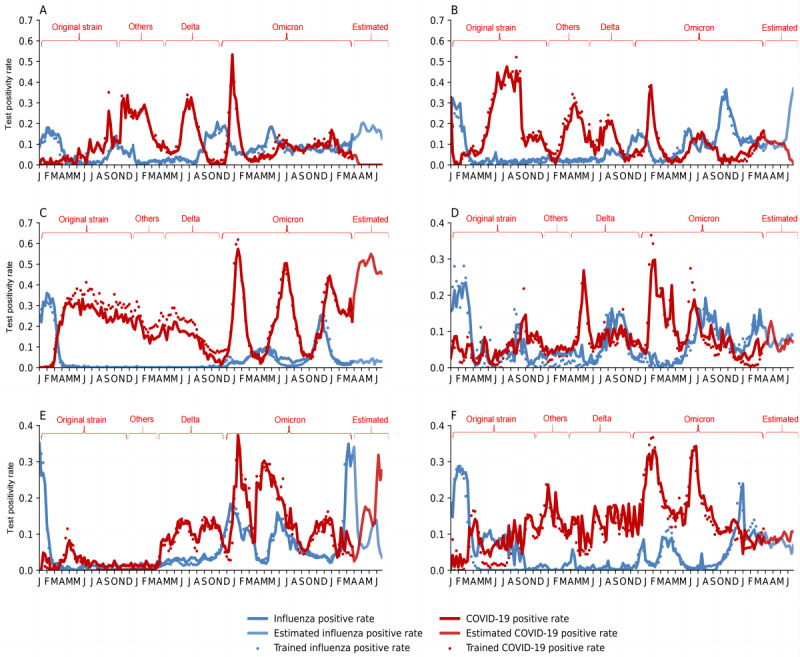
The observed and estimated activity of COVID-19 and influenza. Figures A-F correspond to the following regions of the World Health Organization: African, Eastern Mediterranean, Americas, Southeast Asia, Western Pacific, and European. Periods are set for the original strain (Jan 2020-Nov 2020), “others” (Dec 2020-Apr 2021), Delta (May 2021-Nov 2021), Omicron (Nov 2021-Mar 2023), and the model-estimated period (Mar 2023-July 2023). The horizontal coordinate intervals are divided by month and labeled with the first letter of the month.

During the first 3 months of the Omicron pandemic, influenza activity in the 6 regions first declined alongside soaring Omicron activity and then increased immediately when Omicron plummeted, whereas the influenza test positivity rate overall was consistently lower than that of Omicron. Thereafter, their trends were identical; each declined as the other rose. This occurred 1 to 2 more times, being more apparent in EMRO, AMRO, WPRO, and EURO; each alternation, when influenza activity was higher than Omicron or vice versa, lasted for about 3 to 4 months (Figure 2).

### Estimated COVID-19 and Influenza Trends

We estimated the dynamics of COVID-19 and influenza activity for 16 weeks after the study observation end point (March 26, 2023). The estimation was that they would be alternatingly prevalent at less than 40% positivity rates, except for AMRO (over 40%). In SEARO and EURO, it was estimated that influenza and COVID-19 activity would be closely matched, with both hovering around 10% positivity rates; in AFRO and EMRO, influenza activity would rise from 10% to about 20% to 30%, while COVID-19 activity would decrease from similar levels to less than 5%, and COVID-19 activity would no longer be consistently higher than influenza. Conversely, influenza could decrease to below 10% in AMRO and WPRO as COVID-19 activity might go up to above 30% (Figure 2).

### Correlation Between COVID-19 and Influenza Activity in Different Time Periods

**Figure 3:**
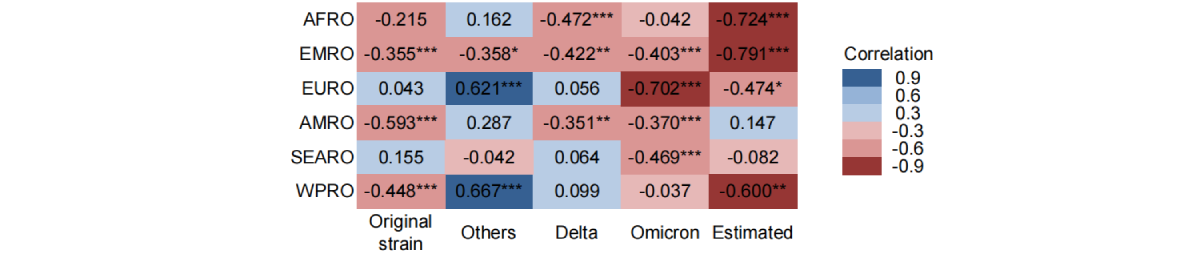
Spearman correlation coefficients for COVID-19 and influenza in different World Health Organization regions. Positive and negative correlations are shown in blue and red, respectively, with darker colors indicating a stronger correlation at the same significance level. AFRO: African region; AMRO: Americas region; EMRO: Eastern Mediterranean region; EURO: European region; SEARO: Southeast Asian region; WPRO: Western Pacific region. *P<.01, **P<.05, ***P<.001.

The Spearman coefficients were calculated for different phases of COVID-19 and influenza activity. In our study, correlation coefficients less than 0.3, 0.3 to less than 0.6, and more than or equal to 0.6 were considered weak, moderate, and strong correlations, respectively. Statistically, COVID-19 and influenza were moderately and negatively correlated in EMRO, AMRO, and WPRO during the original-strain pandemic (–0.355, –0.593, –0.448) and also in EMRO (–0.358) during the multivariant mixed pandemic, while they were transiently, strongly, and positively correlated in EURO and WPRO (0.621 and 0.667). During the Delta pandemic, the 2 diseases were moderately and negatively correlated in AFRO, EMRO, and AMRO (–0.472, –0.422, –0.351). During the Omicron pandemic, they were moderately and negatively correlated in EMRO, AMRO, and SEARO (–0.403, –0.370, –0.469) and highly negatively correlated in EURO (–0.702). Similarly, in the estimated 16-week trend, they showed more significant and highly negative correlations in AFRO, EMRO, and WPRO (–0.724, –0.791, –0.600) and a moderate negative correlation in EURO (–0.474). Overall, COVID-19 and influenza activity were significantly negatively correlated, with coefficients greater than 0.3, especially during the Omicron pandemic and in the upcoming period. No significant correlation was found for other pandemic phases and WHO regions (Figure 3).

## Discussion

### Principal Findings

This study analyzed the dynamics of COVID-19 and influenza in 6 WHO regions over the past 3 years and estimated them in the following 16 weeks. We found that early in the pandemic, when COVID-19 was emerging as an infectious disease, influenza activity stayed below 10% for more than 1 year in the 6 WHO regions. Subsequently, influenza activity gradually rose as Delta activity dropped, but still peaked at a level below that of Delta. Omicron alternated with influenza as the dominant disease. The trend of one disease declining as another rises has been named the seesaw effect [23] and was clearly apparent in the epidemiological patterns of the 2 infectious diseases in this study, characterized by increasing magnitude and frequency, with each alternation have a duration of about 3 to 4 months, suggesting a competitive relationship. The seesaw effect of alternating dominance is likely to become more conspicuous in the postpandemic era. Negative correlation coefficients for different WHO regions and time periods statistically complement the validation of this effect.

Our quantitative analysis based on epidemiological indicators (test positivity rates from the population) showed the dynamic patterns of the 2 respiratory diseases, which have similar transmission modes. The generation and variation of the seesaw effect could be related to numerous factors. On the one hand, from a biological perspective, respiratory viruses share the same host; therefore, viruses compete with each other for susceptible cells in the host. Cells invaded by one respiratory virus produce immune interference that drives uninfected neighboring cells to adopt a protectively antiviral status [24,25], making the host resistant or only partially susceptible to subsequent viruses. One example is the significant decrease in rhinovirus prevalence in patients during peak influenza activity [26]. On the other hand, this effect varies in magnitude and duration across regions, which could be driven by the different intensity of PHSMs adopted in different countries [27-30] and time periods [31], differences in vaccination and natural infection status, or variant strain properties. For example, the earliest adoption of strict nonpharmaceutical interventions in China not only controlled the spread and dissemination of the original strain [32], but also reduced influenza activity by 82%, and by 64% in the north and south [3]. Interestingly, after the PHSMs, influenza activity in China in the latest winter was at a low level because of the rapid rise in Omicron infections in the population at the same time, and after the Omicron test positivity rate dropped, influenza activity jumped sharply, to over 50%. This once again suggests that driving factors influence the magnitude of the seesaw effect and the timing of its onset. In addition, the higher virulence of the original strain attacked individuals more and longer [6], resulting in less opportunity for influenza virus infection; thus, absolute suppression occurred in the first year of the COVID-19 pandemic. The more moderate virulence and lower severity of Delta and Omicron allow the possibility of infecting the host with influenza, which may explain why influenza activity increased after the Delta pandemic and was able to dominate during the Omicron period.

During the multivariant pandemic period, the dynamics of COVID-19 and influenza had short-term positive correlations in EURO and WPRO, while negative correlations were not significant in other regions. Temporary phenomena mediated by complex factors, such as the sudden appearance of variants, require more evidence to unravel their underlying mechanisms. Similar phenomena have been observed in other studies: there are reports of coinfection with SARS-CoV-2 and influenza A in some countries [33-36], and meta-analyses based on the November 2019 to August 2021 period [37] all showed coinfection with both diseases. Nevertheless, most study subjects have been COVID-19 patients or laboratory animals rather than the general population [37,38], with a low proportion of observed coinfections (about 1%) or small sample sizes [39] before and during the Omicron pandemic [37,40]. Inevitably, the positive correlation for susceptibility to coinfection at the individual level or in some high-risk populations might lead to serious clinical outcomes [41]; therefore, much larger, sample-based meta-studies are warranted to explore the reasons for this phenomenon to prevent and control it or to meet the potential surge in hospital visits and demand. In addition, it is worth noting that other respiratory infectious diseases have been threatening humans. Further research on the interactions between respiratory pathogens other than COVID-19 and influenza remains to be done. Regardless of the fate of COVID-19, influenza, or other respiratory pathogens, necessary personal protection and vaccination should be implemented.

Our study provided qualitative and quantitative assessments of the interaction between COVID-19 and influenza. We identified a seesaw effect between COVID-19 and influenza activity based on a competitive effect from an epidemiological perspective, echoing viral antagonism from a historical pathogenic perspective. This finding can be used to guide disease surveillance, early warning, and intervention; for example, real-time surveillance of viral dynamics can be used to estimate trends in other diseases or stagger vaccinations in response to alternating epidemics or pandemics.

### Limitations

There are some limitations to mention. First, the high uncertainty of viral variation, changes in influenza visit behavior triggered by early and rigorous interventions, the decline in SARS-CoV-2 tests in some countries, and other unforeseen factors all interfered with the observed positive test numbers for the 2 diseases studied. Hence, we used the test positivity rate rather than the number of positive tests as an indicator of activity and divided the time periods to minimize the negative impact. Second, the raw data may not fully represent the overall trend in each area because of the limited number of member countries that periodically report to the WHO. Nevertheless, there is no more authoritative data source for COVID-19 and influenza activity.

### Conclusions

Influenza activity and former seasonal epidemiological patterns were shaken by the COVID-19 pandemic. Their activities were inversely correlated to a moderate or greater degree, and they suppressed and competed with each other, showing a seesaw effect. In the postpandemic era of COVID-19, seesaw trends will be more prominent, prompting the possibility of using one disease as an early warning signal for the other for future estimates; this could be used to conduct optimized annual vaccine campaigns.

## Appendix

Multimedia Appendix 1 Supplementary material and tables for the methods and results.

## Abbreviations

AFRO: African region of WHO
AMRO: Americas region of the WHO
EMRO: Eastern Mediterranean region of the WHO
EURO: European region of the WHO
LSTM: long short-term memory
PHSM: public health and social measures
SEARO: Southeast Asia region of the WHO
WHO: World Health Organization
WPRO: Western Pacific region of the WHO

## Data Availability

The data are available in the FluNet repository [10]. The code generated and analyzed during this study are available from the corresponding author on reasonable request.
