# Supplementary material for: Seesaw Effect Between COVID-19 and Influenza From 2020 to 2023 in World Health Organization Regions: Correlation Analysis
Source: JMIR Public Health Surveill. 2023 Jun 12;9:e44970. doi: 10.2196/44970 (PMC10263104; doi:10.2196/44970)
Supplement: Multimedia Appendix 1 [file publichealth_v9i1e44970_app1.docx]

**Appendix**

A seesaw effect between COVID-19 and influenza during 2020-2023 in WHO regions

**Methods and results**

**Data source**

We obtained the test positive rate of the Corona Virus Disease 2019 (COVID-19) and influenza in six regions (Table 1), aiming to explore their epidemiological pattern. The equation for the detection rate (R) was as follows:

$$R(t)=\frac{P(t)}{T(t)}$$

P(t) and T(t) respectively refer to the number of positive tests and number of test samples,

Table 1 Summary of raw data information

| Region* | Indicator | Source | Observed date |
| --- | --- | --- | --- |
| AFRO  EMRO  EURO  AMRO  SEARO  WPRO | - Influenza test positive rate | Reference 10 in the main text | 2020-01-12 to 2023-03-26, weekly |
|  | - COVID-19 test positive rate |  |  |
|  | - Date |  |  |

* Abbreviated regional names: the African Region of WHO (AFRO), Eastern Mediterranean Region of WHO (EMRO), European Region of WHO (EURO), Americas Region of WHO (AMRO). South-East Asia Region of WHO (SEARO) and Western Pacific Region of WHO (WPRO)

**Long short-term memory (LSTM) model parameters selection**

***Proportion selection of training set and testing set***

The training set was used to train the established prediction model to update the parameters. The testing set was used to test the performance of the trained model, which can check the ability to apply to new data and make accurate predictions. In general, the training set should have a higher percentage than the testing set in order to make the built prediction model have a better generalization ability. If the training set is too short, over fitting is likely to occur. When the proportion of the training set to the test set was 6:4, 7:3, 8:2, and 9:1, the performance of the prediction model and the prediction effect were compared as shown in Table 2. According to the results, it showed that the minimum prediction error is reached when the proportion in AFRO, EMRO, EURO, AMRO, SEARO, and WPRO is in the order of 9:1, 9:1, 9:1, 9:1, 8:2, 8:2, and 9:1, respectively.

Table 2 Performance comparison between the training and testing sets with different proportions

|  |  | Proportion | | | |
| --- | --- | --- | --- | --- | --- |
| Region | Indicator | 6：4 | 7：3 | 8：2 | 9：1 |
| AFRO | MAPE | 0.76 | 0.51 | 0.30 | 0.26 |
|  | RMSE | 0.20 | 0.19 | 0.16 | 0.14 |
| EMRO | MAPE | 1.46 | 29.98 | 0.41 | 0.26 |
|  | RMSE | 0.24 | 0.19 | 0.21 | 0.14 |
| EURO | MAPE | 6.69 | 1.73 | 0.53 | 0.27 |
|  | RMSE | 0.23 | 0.20 | 0.19 | 0.17 |
| AMRO | MAPE | 10.21 | 0.80 | 2.72 | 0.46 |
|  | RMSE | 0.24 | 0.19 | 0.23 | 0.19 |
| SEARO | MAPE | 1.34 | 0.91 | 0.40 | 0.54 |
|  | RMSE | 0.19 | 0.17 | 0.18 | 0.19 |
| WPRO | MAPE | 16.52 | 0.57 | 0.52 | 0.92 |
|  | RMSE | 0.29 | 0.25 | 0.23 | 0.24 |

**Selection of steps**

Assuming that the time series data is t=1, 2, .... N, the number t was used to predict the number t+1. t was the step length. Too many steps would lead to gradient disappearance, and too few steps would lead to failure to fully learn the data features. Therefore, the selection of steps plays an important role in the prediction performance. The differences in performance obtained on the testing set with different steps were shown in Table 3.

Table 3 Performance comparison of different steps

| Indicator | Step | AFRO | EMRO | EURO | AMRO | SEARO | WPRO |
| --- | --- | --- | --- | --- | --- | --- | --- |
| MAPE | 1 | 0.73 | 1.14 | 2.25 | 1.06 | 12.57 | 3.6 |
|  | 2 | 0.47 | 1.28 | 0.94 | 0.59 | 0.45 | 1.7 |
|  | 3 | 0.26 | 0.26 | 0.27 | 0.46 | 0.4 | 0.52 |
|  | 4 | 0.25 | 0.2 | 0.27 | 0.33 | 0.46 | 0.72 |
|  | 5 | 0.24 | 0.26 | 0.38 | 0.37 | 3.46 | 0.72 |
|  | 6 | 0.26 | 0.28 | 0.29 | 0.58 | 1.27 | 0.48 |
|  | 7 | 0.27 | 0.19 | 0.44 | 0.83 | 5.79 | 0.43 |
|  | 8 | 0.23 | 0.22 | 1.86 | 1.52 | 0.63 | 0.49 |
| RMSE | 1 | 0.19 | 0.33 | 0.24 | 0.36 | 0.29 | 0.28 |
|  | 2 | 0.23 | 0.21 | 0.2 | 0.22 | 0.2 | 0.38 |
|  | 3 | 0.14 | 0.14 | 0.17 | 0.19 | 0.18 | 0.23 |
|  | 4 | 0.14 | 0.12 | 0.17 | 0.16 | 0.17 | 0.2 |
|  | 5 | 0.14 | 0.15 | 0.2 | 0.18 | 0.19 | 0.28 |
|  | 6 | 0.15 | 0.16 | 0.19 | 0.21 | 0.18 | 0.22 |
|  | 7 | 0.15 | 0.12 | 0.19 | 0.18 | 0.19 | 0.22 |
|  | 8 | 0.14 | 0.14 | 0.21 | 0.18 | 0.17 | 0.25 |

**Selection of layers**

The number of layers affected the accuracy of the model. Increasing the number of layers made the model more capable of learning features, but it also brought problems such as slower convergence, longer computation time, and gradient disappearance between layers. Table 4 showed the performance comparison of prediction models with 1-5 layers.

Table 4 Performance comparison of different layers

|  |  | Layers | | | | |
| --- | --- | --- | --- | --- | --- | --- |
| Region | Indicator | 1 | 2 | 3 | 4 | 5 |
| AFRO | MAPE | 0.20 | 0.20 | 0.23 | 0.28 | 0.24 |
|  | RMSE | 0.13 | 0.13 | 0.14 | 0.17 | 0.14 |
| EMRO | MAPE | 0.21 | 0.26 | 0.19 | 0.24 | 0.28 |
|  | RMSE | 0.13 | 0.15 | 0.12 | 0.14 | 0.16 |
| EURO | MAPE | 3.86 | 0.58 | 0.27 | 0.36 | 0.24 |
|  | RMSE | 0.22 | 0.18 | 0.17 | 0.19 | 0.15 |
| AMRO | MAPE | 0.36 | 0.25 | 0.33 | 1.08 | 0.59 |
|  | RMSE | 0.17 | 0.16 | 0.16 | 0.19 | 0.22 |
| SEARO | MAPE | 0.77 | 1.24 | 0.40 | 1.61 | 1.88 |
|  | RMSE | 0.15 | 0.17 | 0.18 | 0.20 | 0.19 |
| WPRO | MAPE | 0.29 | 0.42 | 0.43 | 0.72 | 0.58 |
|  | RMSE | 0.18 | 0.25 | 0.22 | 0.27 | 0.29 |

**Selection of nodes**

Fewer nodes made the prediction model less capable of processing information, and conversely increased the structural complexity of the prediction model, which also led the convergence slower. In order to find the suitable number of nodes, we calculated the performance in different nodes as shown in Table 5. We found that the best performance was achieved with 128 nodes for all regions except for WPRO with 32 nodes.

Table 5 Performance comparison of different nodes

|  |  | Nodes | | | | |
| --- | --- | --- | --- | --- | --- | --- |
| Region | Indicator | 32 | 64 | 128 | 256 | 512 |
| AFRO | MAPE | 0.23 | 0.24 | 0.20 | 0.21 | 0.23 |
|  | RMSE | 0.15 | 0.14 | 0.13 | 0.13 | 0.13 |
| EMRO | MAPE | 0.29 | 0.39 | 0.19 | 0.24 | 0.29 |
|  | RMSE | 0.17 | 0.16 | 0.12 | 0.15 | 0.16 |
| EURO | MAPE | 0.64 | 0.27 | 0.24 | 1.09 | 0.23 |
|  | RMSE | 0.23 | 0.16 | 0.15 | 0.21 | 0.16 |
| AMRO | MAPE | 0.38 | 0.29 | 0.21 | 0.44 | 0.34 |
|  | RMSE | 0.17 | 0.18 | 0.15 | 0.17 | 0.18 |
| SEARO | MAPE | 0.39 | 0.42 | 0.40 | 1.23 | 0.88 |
|  | RMSE | 0.19 | 0.16 | 0.18 | 0.19 | 0.19 |
| WPRO | MAPE | 0.24 | 0.53 | 0.29 | 0.32 | 0.34 |
|  | RMSE | 0.16 | 0.17 | 0.18 | 0.17 | 0.18 |

**Selection of optimal algorithm**

The optimal algorithm was dependent on continuously adjusting the model bias and weights in order to reduce the value of the loss function and finally achieve convergence. Table 6 showed the prediction results on the testing set using different algorithms (Adam, RMSprop, Adadelta, Adamax, Adagrad). SEARO was more suitable for Adamax, and Adam was preferred for other regions.

Table 6 Performance comparison of different algorithm

|  |  | Algorithm | | | | |
| --- | --- | --- | --- | --- | --- | --- |
| Region | Indicator | Adam | RMSprop | Adadelta | Adamax | Adagrad |
| AFRO | MAPE | 0.20 | 0.25 | 0.33 | 0.20 | 0.66 |
|  | RMSE | 0.13 | 0.15 | 0.17 | 0.14 | 0.15 |
| EMRO | MAPE | 0.19 | 0.24 | 0.57 | 0.31 | 0.31 |
|  | RMSE | 0.12 | 0.14 | 0.24 | 0.17 | 0.17 |
| EURO | MAPE | 0.23 | 1.38 | 1.80 | 1.98 | 0.41 |
|  | RMSE | 0.16 | 0.32 | 0.26 | 0.21 | 0.22 |
| AMRO | MAPE | 0.21 | 0.33 | 0.80 | 1.43 | 3.00 |
|  | RMSE | 0.15 | 0.20 | 0.26 | 0.18 | 0.30 |
| SEARO | MAPE | 0.39 | 3.34 | 0.56 | 0.34 | 1.96 |
|  | RMSE | 0.19 | 0.20 | 0.20 | 0.15 | 0.29 |
| WPRO | MAPE | 0.24 | 0.35 | 0.71 | 0.37 | 0.72 |
|  | RMSE | 0.16 | 0.20 | 0.21 | 0.19 | 0.33 |

In summary, the LSTM model parameters for the six WHO regions were determined and are listed in the table 7.

Table 7 Selected model parameters

| Region | Proportion* | Step | Layer | Node | Algorithm |
| --- | --- | --- | --- | --- | --- |
| AFRO | 9：1 | 8 | 1 | 128 | Adam |
| EMRO | 9：1 | 7 | 3 | 128 | Adam |
| EURO | 9：1 | 4 | 5 | 128 | Adam |
| AMRO | 9：1 | 4 | 2 | 128 | Adam |
| SEARO | 8：2 | 3 | 3 | 128 | Adamax |
| WPRO | 8：2 | 7 | 1 | 32 | Adam |

* Proportion of training set and testing set.
